# Prognostication in patients with idiopathic pulmonary fibrosis using quantitative airway analysis from HRCT: a retrospective study

**DOI:** 10.1183/13993003.00981-2025

**Published:** 2025-10-16

**Authors:** Yang Nan, Federico N. Felder, Stephen Humphries, John A. Mackintosh, Christopher Grainge, Helen E. Jo, Nicole Goh, Paul N. Reynolds, Peter M.A. Hopkins, Vidya Navaratnam, Yuben Moodley, Haydn Walters, Samantha Ellis, Gregory Keir, Chris Zappala, Tamera Corte, Ian Glaspole, Athol U. Wells, Guang Yang, Simon L.F. Walsh

**Affiliations:** 1 Bioengineering Department and Imperial-X, Imperial College London, London, UK; 2 Royal Brompton Hospital, London, UK; 3 National Heart and Lung Institute, Imperial College London, London, UK; 4 Institute of Cardiovascular Science, University College London, London, UK; 5 Prince Charles Hospital, The University of Queensland, Brisbane, Australia; 6 Department of Respiratory Medicine, John Hunter Hospital, Newcastle, Australia; 7 Royal Prince Alfred Hospital, The University of Sydney, Camperdown, Australia; 8 The Austin Hospital, The University of Melbourne, Melbourne, Australia; 9 Royal Adelaide Hospital, The University of Adelaide, Adelaide, Australia; 10 Sir Charles Gairdner Hospital, The University of Western Australia, Nedlands, Australia; 11 Fiona Stanley Hospital, The University of Western Australia, Murdoch, Australia; 12 Allergy and Lung Health Unit, School of Population and Global Health, The University of Melbourne, Melbourne, Australia; 13 Department of Radiology, Alfred Health, Melbourne, Australia; 14 Department of Respiratory Medicine, Princess Alexandra Hospital, Brisbane, Australia; 15 Hervey Bay Hospital, The University of Queensland, Urraween, Australia; 16 NHMRC Centre of Research Excellence in Pulmonary Fibrosis, Sydney, Australia; 17 Respiratory Medicine, Alfred Hospital, Melbourne, Australia; 18 School of Biomedical Engineering and Imaging Sciences, King's College London, London, UK; 19 Equal contribution; 20 Equal senior co-last authors

## Abstract

**Background:**

Predicting shorter life expectancy is crucial for prioritising antifibrotic therapy in fibrotic lung diseases (FLDs), where progression varies widely, from stability to rapid deterioration. This heterogeneity complicates treatment decisions, emphasising the need for reliable baseline measures. This study focuses on leveraging an artificial intelligence (AI) model to address heterogeneity in disease outcomes, focusing on mortality as the ultimate measure of disease trajectory.

**Methods:**

This retrospective study included 1744 anonymised patients who underwent high-resolution computed tomography (HRCT) scanning. The AI model, SABRE (Smart Airway Biomarker Recognition Engine), was developed using data from patients with various lung diseases (n=460, including lung cancer, pneumonia, emphysema and fibrosis). Then, 1284 HRCT scans with evidence of diffuse FLD from the Australian Idiopathic Pulmonary Fibrosis Registry and Open Source Imaging Consortium were used for clinical analyses. Airway branches were categorised and quantified by anatomical structures and volumes, followed by multivariable analysis to explore the associations between these categories and patients’ progression and mortality, adjusting for disease severity or traditional measurements.

**Results:**

Cox regression identified SABRE-based variables as independent predictors of mortality and progression, even adjusting for disease severity (fibrosis extent, traction bronchiectasis extent and interstitial lung disease extent), traditional measures (forced vital capacity percentage predicted, diffusing capacity of the lung for carbon monoxide (*D*_LCO_) percentage predicted and composite physiological index), and previously reported deep learning algorithms for fibrosis quantification and morphological analysis. Combining SABRE with *D*_LCO_ significantly improved prognosis utility, yielding an area under the curve of 0.852 at the first year and a C-index of 0.752.

**Conclusions:**

SABRE-based variables capture prognostic signals beyond that provided by traditional measurements, disease severity scores and established AI-based methods, reflecting the progressiveness and pathogenesis of the disease.

## Shareable abstract

**This paper develops an explainable AI model to extract airway biomarkers for fibrotic lung disease prognostication, capturing prognostic signals above and beyond that provided by traditional measures, disease severity scores and established AI approaches** https://bit.ly/3H0HnL4

## Introduction

Identifying biomarkers that predict shorter life expectancy is essential for prioritising antifibrotic therapy, which can improve outcomes in idiopathic pulmonary fibrosis (IPF) and other fibrotic lung diseases (FLDs) [1–3]. The progression of FLDs varies widely, with some patients remaining stable while others deteriorate rapidly, leading to heterogeneity in outcomes [4]. This variability complicates treatment decisions, including the need for invasive procedures such as surgical lung biopsy, highlighting the importance of reliable baseline measures to predict outcomes. Given that mortality in FLDs reflects life expectancy driven by progression rates, this study focuses on survival as the ultimate end-point, using baseline variables to capture disease progression and heterogeneity.

Over the past decade, there has been a surge in interest surrounding computer-based methods for objectively quantifying FLD on high-resolution computed tomography (HRCT) [5–8]. This heightened interest has been driven by the low reproducibility, high levels of interobserver variability and limited sensitivity of visual HRCT assessments [9]. The severity of traction bronchiectasis, which is the abnormal dilatation of airways in areas of fibrosis, has consistently been reported as a robust predictor of mortality in FLD when quantified visually [10–13]. However, the development of computer-based methods for precise quantification of the severity of traction bronchiectasis has remained elusive. One technical challenge has been accurately distinguishing the airways from areas of honeycombing or emphysema. In this study, we present a computational approach combining an automated deep learning model and a smart quantification algorithm to quantify airway-derived biomarkers and evaluate their prognostic utility in a national IPF registry. The central premise of this study is to leverage this technology to address heterogeneity in disease outcomes, focusing on mortality as the ultimate measure of disease trajectory.

## Methods

### Training SABRE: datasets

SABRE (Smart Airway Biomarker Recognition Engine) development was conducted using ATM22 [14] and AIIB23 [15] data, which were released on Grand Challenge (https://atm22.grand-challenge.org) and Codalab (https://codalab.lisn.upsaclay.fr/competitions/13238) in conjunction with MICCAI 2022 and 2023, respectively. Both ATM22 and AIIB23 datasets were developed to facilitate the advancement of automatic airway modelling tools. ATM22 targets a broad range of pulmonary diseases, including nodules, ground-glass opacities, emphysema and lung cancer, with data from healthy volunteers for broader applicability. In contrast, AIIB23 focuses exclusively on lung fibrosis. Most CT scans have consistent dimensions of 512×512, with few at 768×768, while the number of slices varies (supplementary figure S1). Specifically, ATM22 was annotated by three experienced radiologists (5 years of experience) [14] by delineating the outer margin of the airway branches. AIIB23 (n=50, for testing) and AIPFR data (n=90, for external testing) were annotated by three junior radiologists (2 years of experience) using ITK-SNAP software [16], followed by one experienced radiologist (5 years of experience) and a senior radiologist's revision (10 years of experience). Annotation discrepancies were discussed by senior radiologists and resolved by consensus.

### SABRE architecture

**FIGURE 1:**
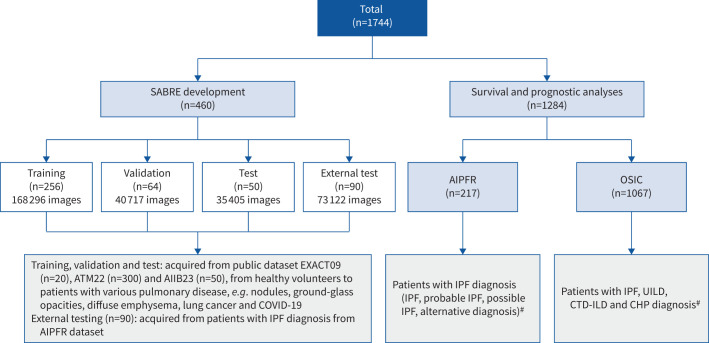
Data distribution of cohorts in this study, with 460 high-resolution computed tomography (HRCT) scans for SABRE development and 1284 scans for survival and prognostic analyses (n-values in the figure represent the number of volumetric HRCT cases). ^#^: based on the 2018 American Thoracic Society/European Respiratory Society/Japanese Respiratory Society/Latin American Thoracic Society guideline [18]. AIPFR: Australian Idiopathic Pulmonary Fibrosis Registry; OSIC: Open Source Imaging Consortium; IPF: idiopathic pulmonary fibrosis; UILD: unclassifiable interstitial lung disease; CTD: connective tissue disease; CHP: chronic hypersensitivity pneumonitis.

**FIGURE 2:**
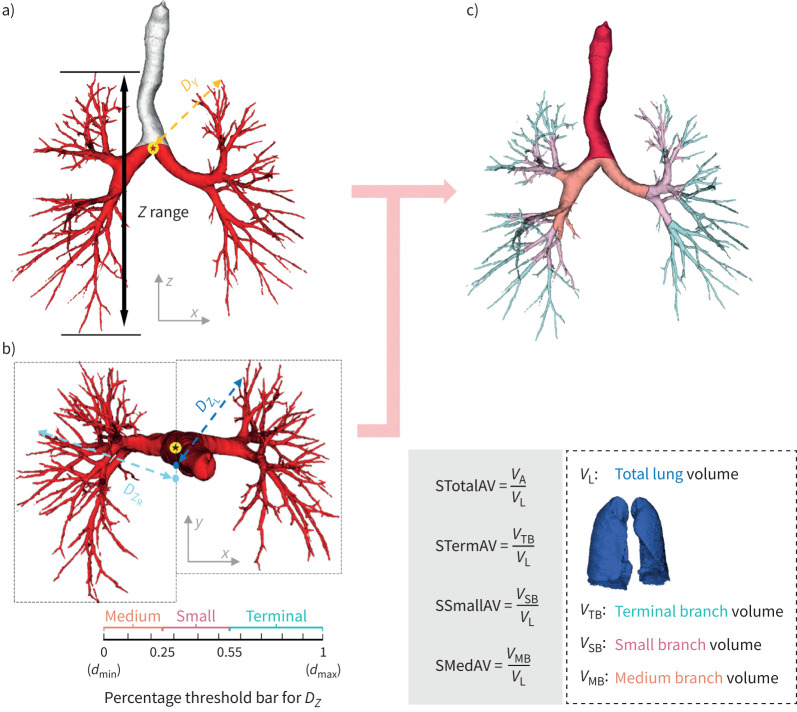
Bronchus modelling based on the distance measurements in a) *y*-axis (sagittal) *P_Y_* and b) *z*-axis (vertical) *P_Z_* projection. The trachea and the spatial centre (yellow stars) are first recognised, followed by estimating the distance between each pixel of airway branches and the adjusted central points (cyan and blue dots) in the *z*-axis projection in b). c) The airway segments are achieved by estimating the height of the airway tree (without trachea) and the percentage distance maps within the left (*D*_Z_L__) and right (*D*_Z_R__) lung regions in the frontal plane and the transverse plane, respectively.

SABRE is an artificial intelligence (AI) system that incorporates a fuzzy attention neural network [17] and a smart branch decomposition module developed (details in the supplementary material) on 587 HRCT cases (figure 1). SABRE estimates airway segments by first localising the trachea and the spatial central point in three-dimensional volumetric data (figure 2). Subsequently, the three-dimensional airway prediction is projected to two two-dimensional images along the *y*-axis (*P_Y_* in figure 2a) and *z*-axis (*P_Z_* in figure 2b). The height of the airway tree (without trachea) is first estimated, followed by calculating the normalised Euclidean distances (range from 0 to 1) between each airway pixel (in *z*-axis projection) and the central point. Based on the distance map, preliminary segments are first set up by thresholds on distance percentiles (the percentiles for recognising terminal, small and medium branches are 0.55 and 0.25, respectively). These preliminary segments are then projected back to three-dimensional volumetric predictions and further revised by the distance constraint of the bronchus trees on *P_Y_*. The pseudo-code for the revised criteria is shown in Algorithm S1 in the supplementary material. Based on these criteria, specific airway segments, namely SABRE-based terminal airway volume (STermAV), small airway volume (SSmallAV), medium airway volume (SMedAV) and total airway volume (STotalAV), are calculated and normalised to total lung volume, resulting in continuous measurements.

### Testing SABRE: Australian Idiopathic Pulmonary Fibrosis Registry and Open Source Imaging Consortium

The clinical utility of SABRE in FLD was evaluated in the Australian Idiopathic Pulmonary Fibrosis Registry (AIPFR; with ethical approval from the Sydney Local Health District, protocol X14-0264) and the Open Source Imaging Consortium (OSIC; www.osicild.org) (table 1). In both registries, patients are referred by their treating physicians with a clinical diagnosis of IPF or other ILDs according to the 2018 American Thoracic Society/European Respiratory Society/Japanese Respiratory Society/Latin American Thoracic Society guidelines [18]. Diagnostic disagreement is resolved by panel discussion, and baseline and longitudinal data are collected for the duration of a participant's enrolment. For the current study, the follow-up period was transplant-free survival, calculated from the date of the registry HRCT.

**TABLE 1:** Baseline demographics and centralised multidisciplinary team meeting review characteristics

	SABRE applicable	SABRE inapplicable
**AIPFR**	n=217	n=298
Age	70.9±8.1	69.6±8.6
Sex		
Male	64.5	69.5
Female	35.5	30.5
Smoking history		
Ever-smoker	152 (74.9)	181 (67.0)
Never-smoker	51 (25.1)	89 (33.0)
Lung function		
FVC % pred (n=204:285)	80.5±22.3	80.3±20.7
*D*_LCO_ % pred (n=173:248)	48.9±16.6	49.2±16.8
Antifibrotic therapy		
No	146 (68.2)	187 (63.2)
Yes	68 (31.8)	109 (36.8)
CPI (n=171:240)	45.1±13.0	45.4±12.9
Fibrosis extent score (n=214:296)	23.9±12.4	22.6±13.1
ILD extent score	34.5±17.1	33.3±17.6
Traction bronchiectasis score (n=214:296)	1.7±0.8	1.7±0.7
UIP diagnosis^#^		
Definite UIP	58 (26.7)	109 (36.6)
Probable UIP	58 (26.7)	78 (26.2)
Indeterminate UIP	66 (30.4)	62 (20.8)
Alternative diagnosis	35 (16.1)	49 (16.4)
**OSIC**	n=1067	n=483
Age	65.0±12.0	66.5±10.3
Sex		
Male	66.3	68.7
Female	33.7	31.3
Smoking history		
Ever-smoker	595 (65.4)	325 (71.0)
Never-smoker	315 (34.6)	133 (29.0)
Lung function		
FVC % pred (n=1059:483)	80.4±21.3	85.1±21.1
*D*_LCO_ (mL·min^−^^1^·mmHg^−1^) (n=604:251)	11.5±5.5	12.5±5.3
FEV_1_ (L) (n=991:474)	2.1±0.7	2.1±0.6
ILD diagnosis		
IPF	530 (53.9)	263 (59.6)
UILD	209 (21.2)	118 (26.8)
CTD-ILD	205 (20.8)	31 (7.0)
CHP	40 (4.1)	29 (6.6)

Data are presented as mean±sd, % or n (%), unless otherwise stated. AIPFR: Australian Idiopathic Pulmonary Fibrosis Registry; IPF: idiopathic pulmonary fibrosis; FVC: forced vital capacity; *D*_LCO_: diffusing capacity of the lung for carbon monoxide; CPI: composite physiological index; ILD: interstitial lung disease; OSIC: Open Source Imaging Consortium; FEV_1_: forced expiratory volume in 1 s; UIP: usual interstitial pneumonia; UILD: unclassifiable interstitial lung disease; CTD: connective tissue disease; CHP: chronic hypersensitivity pneumonitis. ^#^: multidisciplinary team meeting diagnoses were made based on the 2018 American Thoracic Society/European Respiratory Society/Japanese Respiratory Society/Latin American Thoracic Society guideline [18].

In AIPFR, progression at 12 months was defined as a decline in forced vital capacity (FVC) percentage predicted of ≥10% or diffusing capacity of the lung for carbon monoxide (*D*_LCO_) percentage predicted of ≥15%. Two expert radiologists (with 10 and 12 years of experience, respectively [7]) then scored HRCTs for total interstitial lung disease (ILD) extent (ground-glass opacification, reticulation, honeycombing and consolidation), emphysema, total fibrosis and traction bronchiectasis, using definitions and a scoring protocol described in the GitHub online repository (https://github.com/Nandayang/SABRE-prognosis-tool) and supplementary material.

### Statistical analysis

All statistical analyses were performed with Stata 17.0 MP (www.stata.com) and Python SciPy 1.6.2 (https://pypi.org/project/scipy/1.6.2). Data are presented as median with interquartile range (IQR) or mean with standard deviation, depending on distributions, or numbers of patients with percentages where appropriate. Differences between groups were evaluated using the Chi-squared test for categorical variables or a two-sample t-test for parametric continuous variables. The airway modelling performance of SABRE was compared to different methods (nnU-Net [19], WingsNet [20], ERNet [21], ROSE [22], FANN [17], *etc.*) using the Wilcoxon signed-rank test. Linear regression was used to examine relationships between different indices of disease severity and different airway volume indices, adjusting for patient age and sex. Cox proportional hazard modelling was used to determine crude and adjusted hazard ratios. Each unit increase in the SABRE-based variables equivalent to a 0.1% increment in airway branch volume normalised by total lung volume was associated with an *x*-fold increase in risk of mortality. In all multivariable survival analyses, three separate indices were used to control for disease severity: 1) visual-based fibrosis extent (*i.e.* consensed radiologist-based visual score), 2) DeepDTA fibrosis score and 3) composite physiological index (CPI), which is a composite of lung function variables reflecting physiological impairment due to ILD while excluding effects due to emphysema. Since emphysema reduces the severity of traction bronchiectasis [23], separate multivariable survival analyses were performed, controlling for the extent of emphysema. We tested the assumptions of proportional hazards by visual inspection of the log–log plot of survival, comparison of the Kaplan–Meier observed survival curves with the Cox predicted curves for the same variable, and graphical and formal analysis of Schoenfeld residuals. Results are reported as hazard ratios, 95% confidence intervals and p-values. Statistical significance was set at p<0.05.

### SABRE's prognostic utility

Initially, the capability of SABRE in predicting 12-month IPF progression was explored on AIPFR. Then, multivariable analyses were conducted on cohorts stratified by antifibrotic therapy status, excluding patients with short-term mortality to focus on those more likely to be treatment candidates and reduce bias from trial-ineligible individuals. Additionally, the capability of SABRE's risk stratification was investigated. Patients were divided into three groups (low, medium and high risk) based on the tertiles of SABRE-based variables. Kaplan–Meier survival curves were generated for these groups and survival differences were assessed using a log-rank test. To evaluate the performance improvement with SABRE, Harrell's concordance index (C-index) was calculated before and after incorporating SABRE-based primary airway volume (SPAV) into the multivariable analysis, highlighting the model's enhanced discrimination ability. Additionally, time-dependent receiver operating characteristic (ROC) analysis was conducted to evaluate the performance of SABRE and its comparisons.

### Comparison approaches: DeepDTA and AirQuant

Objective fibrosis quantification on HRCT scans was performed using a validated deep learning-based data-driven texture analysis (DeepDTA), *via* convolutional neural networks [24]. DeepDTA was trained on HRCT regions of interest labelled as normal or fibrotic by expert radiologists. For a given scan, the DeepDTA fibrosis score is the percentage of the total lung volume classified as fibrosis. This score has been shown to provide an index of disease severity at baseline with sensitivity to early fibrotic changes as well as progression over time [24]. In addition to DeepDTA, morphological quantification was given by AirQuant [25, 26], an automated computational tool employing a graph network to parcellate airway trees into lung lobes. Airways were split into terminal, small and medium according to their generation percentage thresholds (70% for terminal and small, 40% for small and medium).

## Results

### Clinical parameters, baseline data and analyses

Of 869 AIPFR registrants, 515 with stored HRCT underwent semiquantitative radiological scoring. In parallel, 1550 participants were included from the OSIC registry. Baseline demographics and diagnostic categories for both cohorts are summarised in table 1.

In the AIPFR cohort (n=515), the mean±sd age was 70.1±8.4 years, with the majority being male (347 (67.4%)). The median (IQR) age was 69.7 (64.9–76.1) years. Median (IQR) baseline FVC % pred, *D*_LCO_ % pred and CPI were 79.6% (66.1–93.5%), 46.9% (37.9–58.7%) and 46.1 (36.8–54.8), respectively. Most participants were ever-smokers (64.7%). Median (IQR) follow-up was 4.1 (2.0–6.2) years. 51 had undergone transplants and 328 had died during the follow-up period.

In the OSIC cohort (n=1550), the mean age was 65.4±11.5 years with the majority being male (1040 (67.1%)). Mean±sd baseline FVC % pred and *D*_LCO_ were 81.8±21.3% and 11.8±5.4 mL·min^−^^1^·mmHg^−1^, respectively.. Most participants were also ever-smokers (67.3%) and the majority were diagnosed with IPF (n=793 (55.6%)), unclassifiable ILD (n=327 (22.9%)) or connective tissue disease-associated ILD (n=236 (16.6%)).

For SABRE analysis, patients without volumetric HRCT scans (n=298 in AIPFR and n=483 in OSIC) were excluded, leaving 217 AIPFR and 1067 OSIC patients with scans applicable for analysis. There were no significant differences in baseline characteristics between included and excluded patients. SABRE demonstrated superior performance compared to existing state-of-the-art airway modelling tools (supplementary tables S1 and S2, and supplementary figure S3).

### Univariable analysis of SABRE-based variables

On univariable analysis, all SABRE-based variables predicted mortality and remained predictive when controlling for age and sex (table 2). Relationships between SMedAV, SSmallAV and STermAV were explored. STermAV significantly correlated with SSmallAV on both AIPFR (R^2^=0.69, p<0.0001) and OSIC (R^2^=0.50, p<0.0001) datasets. Given the high degree of collinearity between SSmallAV and STermAV in both cohorts, and the fact that each variable demonstrated independent or near-significant associations with mortality in multivariable analyses (SSmallAV: p=0.001 in OSIC; STermAV: p∼0.05 in both cohorts) (table 2), we elected to combine them into a single metric, SPAV. In bivariable analysis against SMedAV, SPAV outperformed the individual volumes (AIPFR: HR 1.13 (95% CI 1.06–1.20; p<0.0001) and OSIC: HR 1.11 (95% CI 1.07–1.15; p<0.0001), confirming its superior predictive value for mortality.

**TABLE 2:** Results of univariable analysis of individual airway segments, multivariable survival analysis of SABRE-based variables controlled for age and sex, and stepwise analyses for combining STermAV and SSmallAV

	AIPFR (n=217)		OSIC (n=1026)	
	HR (95% CI)	p-value	HR (95% CI)	p-value
**Univariable survival analysis**				
STotalAV	1.13 (1.09–1.17)	<0.0001	1.07 (1.05–1.09)	<0.0001
SMedAV	1.48 (1.30–1.69)	<0.0001	1.12 (1.07–1.17)	<0.0001
SSmallAV	1.42 (1.27–1.58)	<0.0001	1.22 (1.15–1.30)	<0.0001
STermAV	1.26 (1.18–1.34)	<0.0001	1.11 (1.07–1.15)	<0.0001
**Multivariable survival analysis controlled for age and sex**				
STotalAV	1.09 (1.07–1.12)	<0.0001	1.04 (1.02–1.06)	<0.0001
SMedAV	1.44 (1.26–1.65)	<0.0001	1.08 (1.02–1.13)	0.004
SSmallAV	1.40 (1.25–1.56)	<0.0001	1.14 (1.07–1.22)	<0.0001
STermAV	1.25 (1.17–1.33)	<0.0001	1.07 (1.03–1.11)	<0.0001
**Multivariable survival analysis for all airway segments**				
SMedAV	1.14 (0.943–1.39)	0.172	1.05 (0.99–1.11)	0.091
SSmallAV	1.13 (0.929–1.38)	0.221	1.16 (1.07–1.33)	0.001
STermAV	1.13 (0.999–1.27)	0.051	1.06 (1.00–1.13)	0.054
**Multivariable analysis for SMedAV and STermAV**				
SMedAV	1.18 (0.98–1.42)	0.088	1.06 (0.99–1.12)	0.053
STermAV	1.18 (1.08–1.30)	<0.0001	1.13 (1.08–1.19)	<0.0001
**Multivariable analysis for SMedAV and SPAV**				
SMedAV	1.14 (0.94–1.39)	0.172	1.05 (0.99–1.11)	0.114
SPAV	1.13 (1.06–1.20)	<0.0001	1.11 (1.07–1.15)	<0.0001

AIPFR: Australian Idiopathic Pulmonary Fibrosis Registry; OSIC: Open Source Imaging Consortium. See the SABRE architecture section for definitions of SABRE-based variables.

### Multivariable analysis adjusting for lung function measurement

Most SABRE-based variables (STotalAV, SSmallAV, STermAV and SPAV) were independently associated (p<0.001) with mortality against FVC % pred, *D*_LCO_ % pred and CPI, controlled for age and sex on both AIPFR and OSIC (table 3; details in supplementary table S3). Interestingly, the antifibrotic therapy, which impacts progression in IPF independently of disease severity, has broken the link between the airway variable and mortality (supplementary table S4). Specifically, SPAV and STotalAV were highly significant (p<0.0001) in the untreated group, while losing significance in treated patients (p>0.05).

**TABLE 3:** Multivariable survival analyses of SABRE-based variables with lung function measurements or disease severity scores, controlled for age, sex and smoking history

	AIPFR		OSIC (n=1026)	
	HR (95% CI)	p-value	HR (95% CI)	p-value
**With lung function measurements**				
SPAV	1.15 (1.10–1.21)	<0.0001	1.08 (1.04–1.13)	<0.0001
FVC % pred	0.22 (0.08–0.60)	0.003	0.07 (0.04–0.15)	<0.0001
SPAV	1.13 (1.08–1.20)	<0.0001	1.08 (1.03–1.14)	0.004
*D*_LCO_^#^	0.01 (0.002–0.05)	<0.0001	0.89 (0.85–0.93)	<0.0001
STotalAV	1.12 (1.07–1.16)	<0.0001	1.06 (1.03–1.08)	<0.0001
FVC % pred	0.23 (0.09–0.62)	0.004	0.10 (0.05–0.22)	<0.0001
STotalAV	1.10 (1.05–1.14)	<0.0001	1.06 (1.03–1.09)	<0.0001
*D*_LCO_^#^	0.01 (0.002–0.05)	<0.0001	0.90 (0.86–0.94)	<0.0001
**With disease severity scores**				
SPAV	1.11 (1.05–1.18)	<0.0001		
CPI	1.05 (1.03–1.07)	<0.0001		
SPAV	1.12 (1.07–1.18)	<0.0001		
TFE	1.03 (1.02–1.05)	<0.0001		
SPAV	1.13 (1.08–1.20)	<0.0001		
TBX	1.32 (1.00–1.74)	0.048		
SPAV	1.15 (1.10–1.21)	<0.0001		
TIE	1.02 (1.01–1.03)	<0.0001		
STotalAV	1.08 (1.03–1.13)	<0.0001		
CPI	1.05 (1.03–1.07)	<0.0001		
STotalAV	1.10 (1.06–1.14)	<0.0001		
TFE	1.03 (1.01–1.04)	0.001		
STotalAV	1.11 (1.06–1.15)	<0.0001		
TBX	1.31 (1.00–1.73)	0.052		
STotalAV	1.12 (1.08–1.16)	<0.0001		
TIE	1.02 (1.01–1.03)	0.001		

AIPFR: Australian Idiopathic Pulmonary Fibrosis Registry; OSIC: Open Source Imaging Consortium; FVC: forced vital capacity; *D*_LCO_: diffusing capacity of the lung for carbon monoxide; CPI: composite physiological index; TFE: total fibrosis extent; TBX: total traction bronchiectasis extent; TIE: total interstitial lung disease extent. See the SABRE architecture section for definitions of SABRE-based variables. ^#^: we use *D*_LCO_ % pred in AIPFR and *D*_LCO_ in OSIC.

### Multivariable analysis adjusting for disease severity variables

Interobserver agreement for visual-based fibrosis extent was good (intraclass correlation coefficient 0.78 (95% CI 0.76–0.83)). Relationships between visual-based fibrosis extent, automated fibrosis extent (DeepDTA fibrosis score) and CPI were first explored. The results showed that CPI was correlated with visual-based fibrosis extent (R^2^=0.21, p<0.0001) and with DeepDTA fibrosis score (R^2^=0.36, p<0.0001). Additionally, visual-based fibrosis extent was correlated with DeepDTA fibrosis score (R^2^=0.49, p<0.0001) and had associations with extent of traction bronchiectasis (R^2^=0.33, p<0.0001). Correlations between SPAV and visual-based fibrosis extent, DeepDTA fibrosis score and CPI were R^2^=0.25, p<0001, R^2^=0.31, p<0001 and R^2^=0.1, p<0001, respectively (supplementary table S4). Visual-based fibrosis extent and DeepDTA fibrosis score were independently associated with increasing risk of mortality (HR 1.03 (95% CI 1.02–1.04; p<0.0001) and HR 1.03 (95% CI 1.01–1.04; p<0.0001), respectively). Additionally, visual-based traction bronchiectasis (HR 1.42, 95% CI 1.16–1.74; p=0.001) and visual-based fibrosis extent (HR 1.04, 95% CI 1.02–1.05; p<0.0001) were independently associated with mortality.

In multivariable analyses adjusted for age, sex and smoking history (table 4), all SABRE-based variables (full details in supplementary table S5) demonstrated stronger prognostic signal (p<0.0001) compared to visual-based disease severity measures (including total fibrosis extent, total ILD extent and traction bronchiectasis extent). Increasing SPAV score was associated with increasing risk of mortality, independent of disease severity. In all multivariable analyses, controlling for the effect of emphysema (supplementary table S6), the airway segment metrics were empowered (smaller p-value and larger hazard ratio). Similar multivariable analyses of the individual airway segments also demonstrated associations with increasing mortality.

**TABLE 4:** Results of 12-month progression prediction when conducting multivariable analysis with SABRE-based variables, forced vital capacity (FVC) percentage predicted, diffusing capacity of the lung for carbon monoxide (*D*_LCO_) percentage predicted, composite physiological index (CPI) and airway tortuosity, controlled for age, sex and smoking history

	HR (95% CI)	p-value
**STotalAV**	1.10 (1.03–1.17)	0.002
**FVC % pred**	0.48 (0.08–2.95)	0.431
**STotalAV**	1.12 (1.04–1.20)	0.001
***D*_LCO_ % pred**	0.19 (0.02–2.20)	0.182
**STotalAV**	1.12 (1.03–1.21)	0.004
**CPI**	1.02 (0.98–1.05)	0.319
**STotalAV**	1.05 (1.01–1.10)	0.026
**AirQuant tortuosity**	1.19 (1.00–1.41)	0.045
**SPAV**	1.12 (1.05–1.21)	0.002
**FVC % pred**	0.43 (0.07–2.48)	0.344
**SPAV**	1.14 (1.05–1.25)	0.002
***D*_LCO_ % pred**	0.20 (0.02–2.30)	0.194
**SPAV**	1.14 (1.04–1.25)	0.005
**CPI**	1.02 (0.98–1.05)	0.293
**SPAV**	1.09 (1.01–1.18)	0.030
**AirQuant tortuosity**	1.18 (0.99–1.40)	0.059

See the SABRE architecture section for definitions of SABRE-based variables.

### Survival analysis adjusting for airway morphological variables

The association between airway morphological variables (given by AirQuant [25, 26], including sibling angle, length, tortuosity and change in angle) and mortality was first explored. Univariable analyses showed that only tortuosity and angle change for terminal and small branches were independent predictors of mortality (p<0.05). SABRE-based variables revealed stronger association (p<0.001) with mortality when conducting multivariable analysis with tortuosity (p>0.1) and angle change (p>0.01), controlled for age and sex (supplementary table S7).

### Prognostic utility

Both STotalAV and SPAV were independent predictors of 12-month progression when conducting multivariable analysis with FVC % pred, *D*_LCO_ % pred, CPI or airway tortuosity (given by AirQuant), controlled for age, sex and smoking history (table 4). Physiological measures including FVC % pred, *D*_LCO_ % pred and CPI lost their significance (p>0.1) once either STotalAV or SPAV was included when predicting 12-month progression. Interestingly, AirQuant also showed a loss of significance (p=0.045 and 0.059), while both SPAV (HR 1.10, 95% CI 0.99–1.22; p=0.078) and DeepDTA (HR 1.02, 95% CI 0.99–1.04; p=0.088) lost independence for predicting prognosis for multivariable analyses with each other, controlled for age, sex and smoking history. This suggested that these predictive values may be accounted for by STotalAV or SPAV.

**FIGURE 3:**
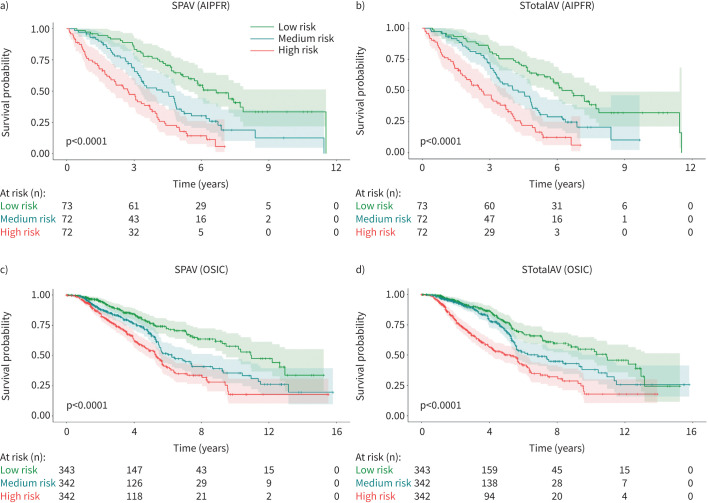
Kaplan–Meier survival curves for low-, medium- and high-risk groups (split by tertiles) based on a, c) SPAV and b, d) STotalAV on a, b) Australian Idiopathic Pulmonary Fibrosis Registry (AIPFR) (n=217) and c, d) Open Source Imaging Consortium (OSIC) (n=1027) datasets. See the SABRE architecture section for definitions of SABRE-based variables.

The Kaplan–Meier survival curves demonstrate a progressive decline in survival across the risk strata (figure 3). Both the medium- and high-risk groups show markedly lower survival compared to the low-risk group, indicating the survival rates decline linearly as SABRE-derived risk levels increase. More importantly, the SABRE-defined high-risk quantile captured 71.1% of 12-month progression events (n=32), whereas the CPI-based high-risk quantile identified only 62.2% (n=28), leaving 38.0% (n=17) of 12-month progression misclassified as low risk. This represents ∼9% gain in sensitivity for SABRE over CPI alone, demonstrating that SABRE not only encompasses the majority of patients flagged by CPI but also reveals an additional subset at elevated risk. These results underscore SABRE's incremental prognostic value and its potential to improve early identification of patients most likely to experience rapid disease progression. Significant differences were also observed between the survival curves of the high-, medium- and low-risk groups (log-rank test p<0.0001) for all SABRE-based variables. For instance, in AIPFR, the 1-year survival rate for the SPAV low-risk group was 97.26% (95% CI 89.49–99.31%) compared to 95.83% (95% CI 87.64–98.64%) for the SPAV medium-risk group. The SPAV high-risk group had a 1-year survival rate of 74.78% (95% CI 62.99–83.30%). Additionally, Cox regression analysis revealed that the HR for the SPAV high-risk group compared to the SPAV low-risk group was 3.84 (95% CI 2.52–5.86; p<0.0001) for AIPFR and 2.54 (95% CI 1.91–3.38; p<0.0001) for OSIC. For the SPAV medium-risk group, the HR compared to the SPAV low-risk group was 2.06 (95% CI 1.35–3.14; p=0.001) for AIPFR and 1.73 (95% CI 1.91–3.38; p<0.0001) for OSIC.

**FIGURE 4:**
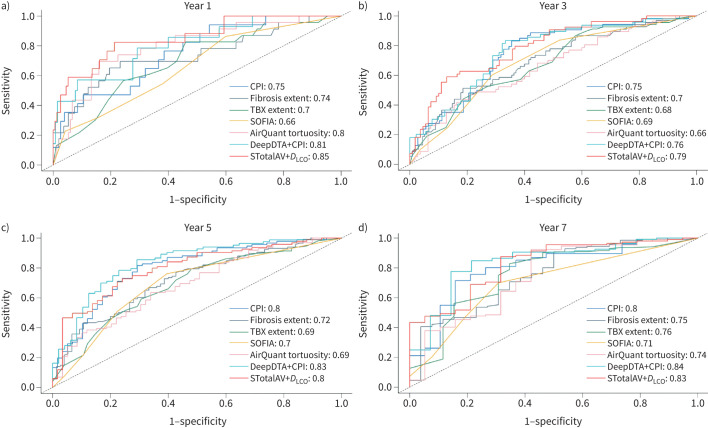
Receiver operating characteristic curves of different models at different time steps: a) year 1, b) year 3, c) year 5 and d) year 7. Area under the curve values are presented. CPI: composite physiological index; TBX: total traction bronchiectasis extent; SOFIA: Systematic Objective Fibrotic Imaging Analysis Algorithm; *D*_LCO_: diffusing capacity of the lung for carbon monoxide. See the SABRE architecture section for definitions of SABRE-based variables.

The inclusion of SABRE-based variables (STermAV, SPAV, SMedAV and SSmallAV) in a multivariable analysis (comprising total fibrosis extent and *D*_LCO_ % pred) resulted in an increment of the C-index from 0.7237 to 0.7541, 0.7551, 0.7409 and 0.7458, respectively. This trend suggests that the SABRE variables contribute additional prognostic information, enhancing the model's ability to differentiate disease severity. Additionally, time-dependent ROC analyses were conducted for different computational models (including SOFIA [3], AirQuant and DeepDTA; details provided in supplementary table S8 and corresponding ROC curves in figure 4). CPI achieved considerable performance (0.777 average area under the curve (AUC) and 0.705 C-index) among traditional measures; fibrosis extent presented the best prognostic utility among disease severity. Notably, AI-based approaches, such as STotalAV and DeepDTA, demonstrated superior prognostic performance compared to traditional measures and disease severity scores. The combination of STotalAV and *D*_LCO_ yielded the highest performance, achieving a C-index of 0.752 and excelling in early stage prediction (year 1) with an impressive AUC of 0.852.

## Discussion

In this study, we demonstrate the prognostic utility of an explainable deep learning model, SABRE, for airway volume quantification in patients with IPF. The model's outcome was independent of disease extent on HRCT and emerged as an independent predictor of 12-month disease progression. The results on AIPFR (n=217) and OSIC (n=1067) showed that SABRE provided additional prognostic information above and beyond that from traditional measures and disease severity.

IPF is typically a progressive FLD with a median untreated survival of 3–5 years, but progression varies widely among individuals: some decline rapidly within months, while others remain stable for years [27]. Similar inexorable progression is observed in progressive pulmonary fibrosis, where antifibrotic therapy has shown benefits in large clinical trials [28, 29]. However, predicting disease progression and monitoring treatment response in FLDs remain challenging, as baseline clinical and imaging data offer limited reliability [30]. While FVC % pred is commonly used for predicting progression, it is prone to variability and missing data, limiting its utility as treatments improve. HRCT, routinely performed in FLD, may provide digital biomarkers to advance precision medicine [18, 31].

Despite more than two decades of evidence supporting the prognostic utility of semiquantitative HRCT evaluation in FLDs, these methods remain underutilised in clinical practice and therapeutic trials. This is due to their inherent subjectivity and high interobserver variability [9], the lack of clarity on how they can effectively guide treatment decisions for individual patients, and their limited sensitivity to marginal disease progression [32]. These challenges have driven the development of computer-based techniques for the objective quantification of FLDs on HRCT. Airway-based models, in particular, have drawn attention given the established link between traction bronchiectasis severity and mortality. However, most existing methods were black-box regressions with limited interpretability, and accurate airway extraction remains challenging in regions affected by honeycombing or emphysema (supplementary figure S2). Our model outperforms the state-of-the-art approaches in airway modelling, and provides superior prognostic utility compared to traditional measurements, radiologist-based visual assessments and existing AI-based tools.

A key finding in this study was the SABRE-based quantitative airway variables were predictors of 12-month progression and mortality. Multivariable analyses revealed that these variables provide valuable prognostic information, independent of visual-assessed disease severity (total fibrosis extent, total traction bronchiectasis extent and total ILD extent) and traditional measures (FVC % pred, *D*_LCO_ % pred and CPI). This observation is consistent with several studies demonstrating that visual grading of traction bronchiectasis severity holds prognostic value not only in IPF but also in fibrotic hypersensitivity pneumonitis, connective tissue disease-related FLD [10–12] and progressive interstitial lung abnormalities [33–35]. Subgroup analyses further demonstrated that SABRE-based variables capture disease progression dynamics and serve as strong independent predictors of 12-month progression adjusted for traditional lung function measures. Moreover, SABRE-based risk stratification effectively identifies patients at higher likelihood of progressing over 12 months. Additionally, the airway signal linked to mortality has been broken by using antifibrotic therapy, suggesting its link to disease pathogenesis. These findings contribute to understanding the underlying pathogenesis of IPF, and highlight the critical role of SABRE in risk stratification, monitoring treatment response and detecting subtle yet clinically meaningful disease progression in FLDs.

SABRE has important implications for clinical trials in IPF and other progressive fibrosing ILDs. One key application is cohort enrichment: by prospectively applying SABRE to screening baseline HRCTs, patients at higher risk of rapid progression can be selected, increasing event rates and enhancing statistical power. Our findings support this strategy, as SABRE-defined high-risk patients had markedly higher 12-month progression rates (71.1%). Incorporating SABRE into screening alongside clinical criteria could therefore optimise trial populations, aligning with the shift toward biomarker-driven enrichment in IPF trials. Additionally, SABRE holds promise as a sensitive imaging end-point. As antifibrotic treatments have reduced the rate of FVC decline, detecting treatment effects based solely on FVC requires larger cohorts or longer follow-up. SABRE-derived airway volumes may reveal structural changes earlier, *e.g.* therapy that stabilises small airways or reduces fibrotic traction may be reflected through SABRE metrics even if FVC decline is minimal. Our findings support this claim, as SABRE variables lost their significance in cohorts that received antifibrotic therapy. Thus, SABRE could serve as a secondary end-point, capturing treatment efficacy not reflected in conventional lung function tests. With further validation, it may eventually qualify as a surrogate end-point, similar to biomarker-based strategies in oncology. Beyond trials, SABRE may inform clinical decisions by enabling earlier detection of progression. Current practice often relies on significant FVC or *D*_LCO_ declines, which may lag behind structural changes. SABRE can detect high-risk changes earlier, *e.g.* a patient with preserved FVC but low SPAV may already be on a steep decline trajectory. Recognising such cases could prompt timely therapy initiation, closer monitoring or transplant evaluation before irreversible damage occurs. While SABRE may not alter treatment eligibility, since antifibrotics are recommended for all IPF patients, it provides prognostic nuance to guide the timing and intensity of care, helping prioritise high-risk individuals for specialist intervention.

When compared with other AI-based tools, SABRE presented higher predictive value. AirQuant-based angle change and tortuosity lost significance when SABRE factors were included in multivariable analyses (supplementary table S7). This suggests that SABRE captures critical aspects of findings that AirQuant cannot fully account for. In time-dependent ROC analysis, while STotalAV demonstrates slightly lower standalone performance compared to DeepDTA, its clinical utility lies in its superior interpretability and flexibility. Importantly, STotalAV with *D*_LCO_ achieves the best overall performance (especially at the early stage) among all established approaches, underscoring its capacity to enhance prognostic accuracy in a clinically meaningful way.

Our study raises an interesting question regarding what prognostic signal the airways capture. Since SABRE-based variables correspond to normalised airway volumes, a possible explanation is that these variables reflect the progressiveness of fibrosis in addition to severity; a hypothesis supported by a study that has linked severity of traction bronchiectasis (the airway dilation) to the profusion of fibroblastic foci on surgical lung biopsy in IPF patients [36]. Fibroblastic foci represent active zones of collagen formation and deposition, and have also been linked with mortality. This has important clinical implications; while the association between extensive fibrosis on HRCT and mortality in FLDs is established, predicting future disease progression in patients early in their disease course when fibrosis is less extensive on HRCT remains challenging. Our findings demonstrate that SABRE-based variables not only reflect traction bronchiectasis but surpass it as an even more robust independent predictor of mortality adjusting for disease extent. This suggests SABRE could help stratify patients with limited disease, enabling earlier intervention with antifibrotic treatments before significant lung function decline has occurred. Another notable finding was the prognostic value of proximal airways, typically considered less relevant in IPF. This may reflect initial damage to medium airways caused by environmental or mechanical insults years before IPF onset, aligning with the “two-hit” hypothesis. Exposures such as wood dust, industrial pollutants, smoking or micro-aspiration of gastric acid could have left residual impacts on these airways before disease progression.

Generalisability is crucial for integrating deep learning models into clinical practice. A key strength of this study is the use of independent cohorts from a national IPF registry and the largest open-source dataset, featuring well-characterised FLD patients and CT scans obtained from various scanners with diverse acquisition protocols. Despite this heterogeneity, SABRE demonstrated robust performance and clinical applicability. However, SABRE relies on volumetric HRCT data, which limits its application to interspaced CT scans, a common practice in many centres. This highlights the need for global standardisation of HRCT protocols to ensure consistent data quality and support the adoption of advanced computer-based analysis methods. Future research should explore automated longitudinal evaluation of airway volume changes, as previous studies have shown that changes in traction bronchiectasis severity scores are sensitive markers of progression in IPF patients with marginal FVC decline. Additionally, challenges remain in bronchial segmentation within regions of extensive honeycombing, requiring further advancements in network architecture, data quantity and training protocols.

In conclusion, we have demonstrated the prognostic utility of SABRE, a deep learning-based model for quantifying airways in IPF patients enrolled in a national IPF registry. The model captures prognostic signals above and beyond that provided by traditional disease severity scores and clinical measures, possibly reflecting the progressiveness and pathogenesis of disease. In principle, this tool could be used alongside other biomarkers such as lung function or proteomic data to identify FLD patients at risk of progression and for patient stratification in clinical trials.

## Shareable PDF

10.1183/13993003.00981-2025.Shareable1 This PDF extract can be shared freely online.
Shareable PDF

ERJ-00981-2025.Shareable

## Data Availability

Individual participant data (including data dictionaries) will not be made available. Links for publicly available data (HRCT scans) and analytic code will be shared within 3 months of publication of this work, with anyone who wishes to have access. Data may not be used for commercial purposes. Codes are available at https://github.com/Nandayang/IPF-prognosis/tree/main

## Supplementary material

**Please note:** supplementary material is not edited by the Editorial Office, and is uploaded as it has been supplied by the author. Supplementary file: appendix ERJ-00981-2025.Supplement
