# Supplementary material for: Prognostication in patients with idiopathic pulmonary fibrosis using quantitative airway analysis from HRCT: a retrospective study
Source: Eur Respir J. 2025 Oct 16;66(4):2500981. doi: 10.1183/13993003.00981-2025 (PMC12528776; doi:10.1183/13993003.00981-2025)
Supplement: Supplementary file 1 [file ERJ-00981-2025.Shareable.pdf]

EUROPEAN RESPIRATORY JOURNAL
ORIGINAL RESEARCH ARTICLE
Y. NAN ET AL.

SHAREABLE PDF

Prognostication in patients with idiopathic pulmonary fibrosis using quantitative airway analysis from HRCT: a retrospective study

Yang Nan[1,2,19], Federico N. Felder[2,3,19], Stephen Humphries[4], John A. Mackintosh[5], Christopher Grainge[6], Helen E. Jo[7], Nicole Goh[8], Paul N. Reynolds[9], Peter M.A. Hopkins[5], Vidya Navaratnam[10], Yuben Moodley[11], Haydn Walters[12], Samantha Ellis[13], Gregory Keir[14], Chris Zappala[15], Tamera Corte[7,16], Ian Glaspole[17], Athol U. Wells[2,3], Guang Yang[1,2,3,18,20] and Simon L.F. Walsh[2,3,20]

[1]Bioengineering Department and Imperial-X, Imperial College London, London, UK. [2]Royal Brompton Hospital, London, UK. [3]National Heart and Lung Institute, Imperial College London, London, UK. [4]Institute of Cardiovascular Science, University College London, London, UK. [5]Prince Charles Hospital, The University of Queensland, Brisbane, Australia. [6]Department of Respiratory Medicine, John Hunter Hospital, Newcastle, Australia. [7]Royal Prince Alfred Hospital, The University of Sydney, Camperdown, Australia. [8]The Austin Hospital, The University of Melbourne, Melbourne, Australia. [9]Royal Adelaide Hospital, The University of Adelaide, Adelaide, Australia. [10]Sir Charles Gairdner Hospital, The University of Western Australia, Nedlands, Australia. [11]Fiona Stanley Hospital, The University of Western Australia, Murdoch, Australia. [12]Allergy and Lung Health Unit, School of Population and Global Health, The University of Melbourne, Melbourne, Australia. [13]Department of Radiology, Alfred Health, Melbourne, Australia. [14]Department of Respiratory Medicine, Princess Alexandra Hospital, Brisbane, Australia. [15]Hervey Bay Hospital, The University of Queensland, Urraween, Australia. [16]NHMRC Centre of Research Excellence in Pulmonary Fibrosis, Sydney, Australia. [17]Respiratory Medicine, Alfred Hospital, Melbourne, Australia. [18]School of Biomedical Engineering and Imaging Sciences, King's College London, London, UK. [19]Equal contribution. [20]Equal senior co-last authors.

Corresponding author: Yang Nan (y.nan20@imperial.ac.uk)

Shareable abstract (@ERSpublications)
This paper develops an explainable AI model to extract airway biomarkers for fibrotic lung disease prognostication, capturing prognostic signals above and beyond that provided by traditional measures, disease severity scores and established AI approaches https://bit.ly/3H0HnL4

Cite this article as: Nan Y, Felder FN, Humphries S, *et al*. Prognostication in patients with idiopathic pulmonary fibrosis using quantitative airway analysis from HRCT: a retrospective study. *Eur Respir J* 2025; 66: 2500981 [DOI: 10.1183/13993003.00981-2025].

This PDF extract can be shared freely online.

Copyright ©The authors 2025

This version is distributed under the terms of the Creative Commons Attribution Licence 4.0.

This article has an editorial commentary: https://doi.org/10.1183/13993003.01516-2025

Received: 1 May 2024
Accepted: 1 June 2025

Abstract

Background Predicting shorter life expectancy is crucial for prioritising antifibrotic therapy in fibrotic lung diseases (FLDs), where progression varies widely, from stability to rapid deterioration. This heterogeneity complicates treatment decisions, emphasising the need for reliable baseline measures. This study focuses on leveraging an artificial intelligence (AI) model to address heterogeneity in disease outcomes, focusing on mortality as the ultimate measure of disease trajectory.

Methods This retrospective study included 1744 anonymised patients who underwent high-resolution computed tomography (HRCT) scanning. The AI model, SABRE (Smart Airway Biomarker Recognition Engine), was developed using data from patients with various lung diseases (n=460, including lung cancer, pneumonia, emphysema and fibrosis). Then, 1284 HRCT scans with evidence of diffuse FLD from the Australian Idiopathic Pulmonary Fibrosis Registry and Open Source Imaging Consortium were used for clinical analyses. Airway branches were categorised and quantified by anatomical structures and volumes, followed by multivariable analysis to explore the associations between these categories and patients' progression and mortality, adjusting for disease severity or traditional measurements.

Results Cox regression identified SABRE-based variables as independent predictors of mortality and progression, even adjusting for disease severity (fibrosis extent, traction bronchiectasis extent and interstitial lung disease extent), traditional measures (forced vital capacity percentage predicted, diffusing capacity of the lung for carbon monoxide (D_{LCO}) percentage predicted and composite physiological index), and previously reported deep learning algorithms for fibrosis quantification and morphological analysis.

Combining SABRE with D_{LCO} significantly improved prognosis utility, yielding an area under the curve of 0.852 at the first year and a C-index of 0.752.

Conclusions SABRE-based variables capture prognostic signals beyond that provided by traditional measurements, disease severity scores and established AI-based methods, reflecting the progressiveness and pathogenesis of the disease.
