# Supplementary material for: Prognostication in patients with idiopathic pulmonary fibrosis using quantitative airway analysis from HRCT: a retrospective study
Source: Eur Respir J. 2025 Oct 16;66(4):2500981. doi: 10.1183/13993003.00981-2025 (PMC12528776; doi:10.1183/13993003.00981-2025)
Supplement: Supplementary file 2 [file ERJ-00981-2025.Supplement.pdf]

Appendix

Supplementary Figures

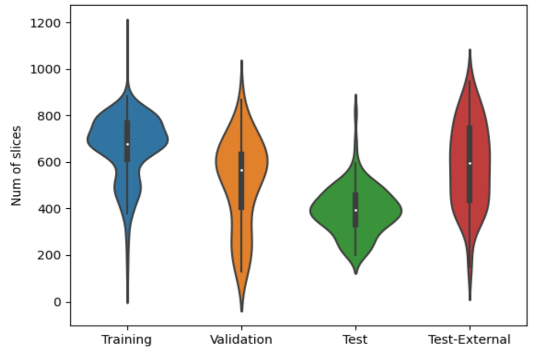

Figure S1. Variation in the number of slices of different sets in this study.

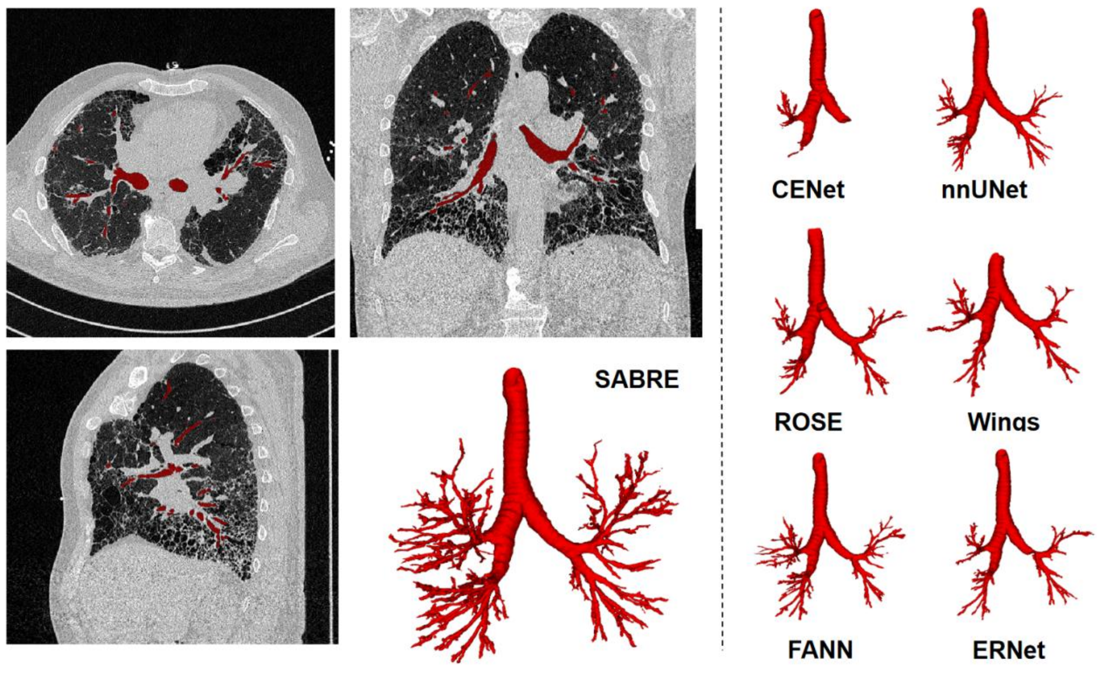

Figure S2. Visualization results of SABRE and its comparisons.

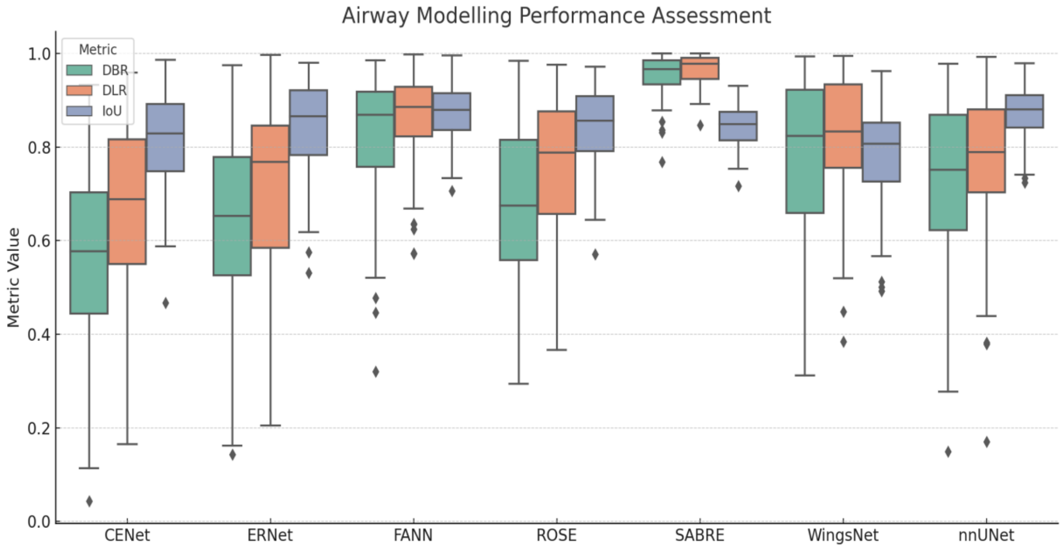

Figure S3. Box plot illustrating the Intersection over Union (IoU) score, Detected Branch Ratio (DBR), and Detected Length Ratio (DLR) for airway modelling performance across different approaches.

Supplementary Tables

Models	IoU	DLR	DBR
nnUNet	0.883±0.049†	0.748±0.179†	0.688±0.204†
WingsNet	0.786±0.085†	0.810±0.143†	0.768±0.172†
ERNet	0.845±0.096	0.704±0.189†	0.638±0.199†
ROSE	0.852±0.083*	0.755±0.156†	0.687±0.169†
CENet	0.824±0.104†	0.639±0.204†	0.563±0.204†
FANN	0.875±0.058†	0.861±0.099†	0.818±0.120†
SABRE	0.848±0.041	0.959±0.037	0.943±0.056

Table S1. Segmentation performance of SABRE (n=90), † indicates highly significant differences (p<0.001) and * refers to significant (p<0.05) evaluated by Wilcoxon signed-rank test, IoU refers to the intersection of union, DLR and DBR indicate detected length ratio and detected branch ratio for airway trees, respectively.

Cohorts	Models	IoU	DLR	DBR
	nnUNet	0.907±0.042*	0.917±0.074†	0.876±0.088†
	WingsNet	0.835±0.061†	0.918±0.072†	0.894±0.095†
	ERNet	0.892±0.057	0.833±0.089†	0.787±0.112†
COVID-19	ROSE	0.912±0.034†	0.849±0.081†	0.772±0.094†
	CENet	0.889±0.053	0.772±0.136†	0.669±0.153†
	FANN	0.914±0.028*	0.931±0.063†	0.905±0.776†
	SABRE	0.896±0.050	0.974±0.022	0.971±0.031
	nnUNet	0.869±0.051†	0.722±0.159†	0.673±0.197†
	WingsNet	0.773±0.092†	0.794±0.138†	0.755±0.180†
	ERNet	0.856±0.088	0.690±0.155†	0.604±0.192†
Fibrosis	ROSE	0.838±0.095*	0.731±0.164†	0.668±0.163†
	CENet	0.812±0.116†	0.625±0.210†	0.560±0.198†
	FANN	0.869±0.059*	0.841±0.087†	0.802±0.109†
	SABRE	0.851±0.048	0.962±0.041	0.944±0.060

Table S2. Segmentation performance of SABRE and its comparisons on different cohorts, † indicates highly significant differences (p<0.001) and * refers to significant (p<0.05) evaluated by Wilcoxon signed-rank test. IoU refers to intersection of union, DLR and DBR indicate detected length ratio and detected branch ratio for airway trees, respectively.

Variable	HR	P Value	CI 95%
STermAV	1.20	<0.0001	1.12-1.28
FVC%	0.18	<0.0001	0.07-0.47
STermAV	1.19	<0.0001	1.10-1.28
DLCO%	0.01	<0.0001	0.00-0.04
STermAV	1.15	0.001	1.06-1.25
CPI	1.05	<0.0001	1.04-1.07
SSmallAV	1.34	<0.0001	1.19-1.50
FVC%	0.16	<0.0001	0.06-0.39
SSmallAV	1.31	<0.0001	1.15-1.48
DLCO%	0.01	<0.0001	0.00-0.03
SSmallAV	1.26	<0.0001	1.11-1.44
CPI	1.06	<0.0001	1.04-1.08
SMedAV	1.34	<0.0001	1.16-1.54
FVC%	0.17	<0.0001	0.07-0.45
SMedAV	1.21	0.012	1.04-1.40
DLCO%	0.01	<0.0001	0.00-0.03
SMedAV	1.16	0.058	0.99-1.36
CPI	1.06	<0.0001	1.04-1.08
SPAV	1.13	<0.0001	1.08-1.19
FVC%	0.18	<0.0001	0.07-0.46
SPAV	1.12	<0.0001	1.07-1.18
DLCO%	0.01	<0.0001	0.00-0.04
SPAV	1.10	<0.0001	1.05-1.16
CPI	1.05	<0.0001	1.04-1.07
STotalAV	1.10	<0.0001	1.06-1.14
FVC%	0.19	0.001	0.07-0.49

Variable	HR	P Value	CI 95%
STotalAV	1.09	<0.0001	1.05-1.13
DLCO%	0.01	<0.0001	0.00-0.04
STotalAV	1.07	0.001	1.03-1.12
CPI	1.05	<0.0001	1.04-1.07

Table S3. Multivariable survival analyses of SABRE-based volumes with traditional measurement FVC%, DLCO%, and CPI, respectively, controlled for age and sex.

Variable	Total fibrosis extent (n=214)	DeepDTA score (n=164)	CPI (n=171)
STotalAV	R^2=0.26, p<0.0001	R^2=0.32, p<0.0001	R^2=0.14, p<0.0001
SMedAV	R^2=0.17, p<0.0001	R^2=0.21, p<0.0001	R^2=0.16, p<0.0001
SSmallAV	R^2=0.21, p<0.0001	R^2=0.22, p<0.0001	R^2=0.09, p=0.0001
STermAV	R^2=0.25, p<0.0001	R^2=0.32, p<0.0001	R^2=0.11, p<0.0001
SPAV	R^2=0.25, p<0.0001	R^2=0.31, p<0.0001	R^2=0.11, p<0.0001

Table S4. Relationship between disease severity (using total fibrosis extent scores, DeepDTA score and the CPI) and SABRE-based airway variables.

Variable	Without antifibrotic treatment (n=100)			With antifibrotic treatment (n=61)		
	**HR**	**P Value**	**CI 95%**	**HR**	**P Value**	**CI 95%**
STermAV	1.25	<0.0001	1.11-1.41	1.12	0.127	0.97-1.29
DLCO%	0.01	<0.0001	0.00-0.10	0.01	<0.0001	0.00-0.12
SSmallAV	1.31	0.002	1.11-1.54	1.29	0.05	1.00-1.66
DLCO%	0.01	<0.0001	0.00-0.09	0.01	<0.0001	0.00-0.10
SMedAV	1.35	0.027	1.03-1.77	1.17	0.234	0.90-1.53
DLCO%	0.01	<0.0001	0.00-0.11	0.01	<0.0001	0.00-0.11
SPAV	1.15	<0.0001	1.06-1.23	1.11	0.070	0.99-1.21
DLCO%	0.01	<0.0001	0.00-0.10	0.01	<0.0001	0.00-0.12
STotalAV	1.13	<0.0001	1.05-1.18	1.07	0.086	0.99-1.15
DLCO %	0.01	<0.0001	0.00-0.10	0.01	0.001	0.00-0.13

Table S5. Multivariable survival analyses (excluding short term mortality patients) of SABRE-based variables with DLCO%, controlled for age and sex in patients with/without antifibrotic treatment. The airway linked to mortality has been broken by using antifibrotic therapy, suggesting its link to disease pathogenic.

Variable	Without Emphysema(n=91)			With Emphysema(n=123)		
	HR	P Value	CI 95%	HR	P Value	CI 95%
STermAV (n=164)	1.15	0.001	1.06-1.25	1.19	<0.0001	1.10-1.30
CPI	1.05	<0.0001	1.04-1.07	1.05	<0.0001	1.03-1.07
STermAV (n=162)	1.10	0.051	1.00-1.20	1.13	0.01	1.03-1.24
DeepDTA fibrosis score	1.03	<0.0001	1.02-1.04	1.03	<0.0001	1.01-1.04
STermAV (n=214)	1.16	<0.0001	1.07-1.25	1.18	<0.0001	1.10-1.27
Total fibrosis extent	1.04	<0.0001	1.02-1.05	1.04	<0.0001	1.02-1.06
SSmallAV (n=164)	1.26	<0.0001	1.11-1.44	1.36	<0.0001	1.19-1.56
CPI	1.06	<0.0001	1.04-1.08	1.06	<0.0001	1.04-1.07
SSmallAV (n=162)	1.23	0.007	1.06-1.43	1.33	<0.0001	1.14-1.56
DeepDTA fibrosis score	1.03	<0.0001	1.02-1.04	1.03	<0.0001	1.02-1.04
SSmallAV (n=214)	1.26	<0.0001	1.12-1.43	1.34	<0.0001	1.18-1.53
Total fibrosis extent	1.4	<0.0001	1.02-1.06	1.04	<0.0001	1.02-1.06
SMedAV (n=164)	1.16	0.058	0.99-1.36	1.24	0.008	1.06-1.46
CPI	1.06	<0.0001	1.04-1.08	1.56	<0.0001	1.04-1.08
SMedAV (n=162)	1.08	0.382	0.91-1.27	1.15	0.106	0.97-1.37
DeepDTA fibrosis score	1.03	<0.0001	1.02-1.05	1.03	<0.0001	1.02-1.04
SMedAV (n=214)	1.28	0.001	1.11-1.48	1.37	<0.0001	1.19-1.59
Total fibrosis extent	1.04	<0.0001	1.03-1.06	1.04	<0.0001	1.03-1.06
SPAV (n=164)	1.10	<0.0001	1.05-1.16	1.14	<0.0001	1.07-1.20
CPI	1.05	<0.0001	1.04-1.07	1.05	<0.0001	1.03-1.07
SPAV (n=162)	1.07	0.019	1.01-1.14	1.10	0.002	1.04-1.17
DeepDTA fibrosis score	1.03	<0.0001	1.02-1.04	1.03	<0.0001	1.01-1.04
SPAV (n=214)	1.11	<0.0001	1.05-1.16	1.13	<0.0001	1.07-1.18
Total fibrosis extent	1.04	<0.0001	1.02-1.05	1.04	<0.0001	1.02-1.05

Table S6. Multivariable survival analysis of each of SABRE-based airway segment volumes with disease severity controlled for age and sex.

Variable	HR	P Value	CI 95%
STermAV	1.20	<0.0001	1.11-1.30
Term_Angle_Change	1.00	0.888	0.95-1.06
Term_Tortuosity	1.11	0.120	0.97-1.26
SSmallAV	1.26	0.004	1.08-1.48
Small_Angle_Change	1.08	0.029	1.00-1.17
Small_Tortuosity	1.18	0.138	0.95-1.45
SMedAV	1.41	<0.0001	1.21-1.65
Medium_Angle_Change	1.00	0.989	0.94-1.06
Medium_Tortuosity	1.09	0.117	0.98-1.22

Table S7. Multivariable survival analysis of SABRE-based volumes with airway morphological variables (given by AirQuant [1]), controlled for age and sex.

Variables	AUC Y1	AUC Y3	AUC Y5	AUC Y7	Avg AUC	C-index
DLCO	0.211	0.253	0.245	0.241	0.237	0.670
FVC	0.210	0.361	0.394	0.380	0.336	0.584
CPI	0.754	0.752	0.799	0.802	0.777	0.705
Fibrosis extent	0.736	0.699	0.721	0.752	0.727	0.676
Tbx extent	0.699	0.677	0.691	0.757	0.706	0.652
ILD extent	0.774	0.677	0.659	0.695	0.701	0.655
DeepDTA	0.840	0.756	0.766	0.833	0.799	0.713
SOFIA	0.660	0.693	0.695	0.705	0.688	0.646
AirQuant-tor	0.798	0.660	0.692	0.745	0.724	0.643
AirQuant-ang	0.822	0.739	0.614	0.638	0.703	0.640
STotalAV	0.829	0.754	0.741	0.825	0.787	0.683
DeepDTA +DLCO	0.826	0.767	0.783	0.818	0.798	0.728
STotalAV+DLCO	**0.852**	**0.788**	0.799	0.826	**0.816**	**0.752**
DeepDTA +CPI	0.805	0.761	**0.833**	0.837	0.809	0.727
STotalAV+CPI	0.817	0.783	0.821	**0.852**	0.818	0.740

Table S8. Area under the curve (AUC) of traditional measures, disease severity scores, and AI-derived variables at year 1 (Y1), year 3 (Y3), year 5 (Y5), year 7 (Y7), and Concordance Index. Tbx refers to traction bronchiectasis, the AirQuant-tor and AirQuant-ang refer to tortuosity and angle change given by AirQuant, respectively.

Semiquantitative HRCT evaluation

Each HRCT scan was scored independently by two thoracic radiologists (LC, MS, 10- and 12-years' experience) who were blinded to all clinical information. HRCTs were scored on a lobar basis. The total extent of interstitial lung disease (ILD) was initially estimated to the nearest 5%, then subclassified into four patterns: ground glass opacification, reticulation, honeycombing, consolidation, and emphysema, using definitions from the Fleischner Society glossary of terms for thoracic imaging. Parenchymal pattern scores for each lobe were generated by multiplying the total lobar ILD extent by the individual lobar parenchymal pattern extents and dividing by 100. The individual lobar percentages of each parenchymal pattern were summed for each radiologist and a total extent score for each pattern, was for each HRCT. Traction bronchiectasis, as defined in the Fleischner Society glossary of terms, was assigned a severity score (none:0, mild:1, moderate:2, severe:3) for each lobe and these scores were summed to give a total traction bronchiectasis severity score for each HRCT, for each radiologist. Average total ILD extent scores, total parenchymal pattern scores and total traction bronchiectasis severity scores were generated for each HRCT from the individual radiologists' scores.

Each radiologist provided a 0-100% probability score for each of the four ATS/ERS/JRS/LATS 2018 guideline categories (definite UIP, probable UIP, indeterminate for UIP, alternative diagnosis), summating to 100% e.g., UIP:75%, probable UIP 25%, indeterminate for UIP: 0%, alternative diagnosis:0%. Average diagnosis category probabilities for each HRCT were generated from the individual radiologists' scores. The final first-choice diagnosis for each HRCT was taken as the diagnosis category with the highest probability. Consensus was reached for cases where the probability of two diagnosis categories were equal e.g., UIP:50%, probable UIP 50%, indeterminate for UIP: 0%, alternative diagnosis:0%.

SABRE development

For model development, 370 CT scans from ATM22 and AIIB23 were split as train (n=256), validate (n=64) and test (n=50). To test the model's segmentation capacity and robustness, 90 scans of patients with FLDs were used as the external test set. Given the intensive computational requirements of training volumetric HRCT scans, SABRE was trained on volumetric patches

(extracted from raw HRCT scan) and employed the 'split-merge' strategy during the inference stage. Specifically, HRCT with their annotations were first split into volumetric patches, followed by the combination of patch predictions. We used the smart patch sampling strategy in our preliminary study [2] to extract patches (for training). In particular, the average shapes of minimum bounding boxes of the airway tree annotations (from human experts) were summarized to define the size of volumetric patches. Then, airway centrelines were extracted through skeletonization algorithm. Patches with a centreline ratio greater than 15% were included to training sets, this aims to alleviate the data-imbalanced issues between the airways and lung tissues. Additionally, z-score normalization was implemented to all volumetric patches x by

$$x' = \frac{x - \mu}{\sigma},$$

where μ and σ are the average mean and standard deviation values of the airway voxels in the training data. It is of note that for the test stage, all patches were normalized by the same mean μ and standard deviation σ. To improve the consistency and continuity of airway predictions, an optimization function that incorporates the airway centreline was included. The entire system was developed through Pytorch 1.10, integrated with CUDA 11.1.74 and cuDNN 8.0.5, operating on an Ubuntu 20.04.2 LTS platform with the NVIDIA RTX 3090.

Randomized vertical/horizontal flips were simultaneously implemented to input image patches and their corresponding annotations for data augmentation during the training. The model was trained for 200 epochs for convergence through Adam optimizer (with the initial learning rate of and the decay rate of 0.95 for each epoch). All the trainable parameters of the neural network were initialized by He initialization [3].

Evaluation metrics of SABRE

Given the airway prediction mask X and the expert annotation Y, the evaluation metrics of SABRE include the Intersection over union (IoU) score, detected length rate (DLR), branch rate (DBR).

The IoU score assess the overlap ratio between the clinicians' annotations and the output of AI model, denoted as:

$$\mathrm{IoU} = \frac{XY}{X + Y - XY},$$

where XY is the intersection of X and Y.

DBR and DLR assess the predictions in terms of the detected length L_X and branches N_X

$$DBR = \frac{N_X}{N_Y},$$

$$DLR = \frac{L_X}{L_Y},$$

where N_Y and L_Y are the total number and total length of airway branches given by expert annotation, respectively. Specifically, we consider an airway branch to be correctly detected if its IoU score exceeds 0.8 when compared with the clinicians' annotation.

Model Performance of SABRE

The performance of SABRE and its comparisons are shown in Table S1 and Figure S3. In particular, SABRE achieved the best performance on branch completeness and continuity, with an average detected branch ratio of 0.943 $\pm$ 0.056, and an average detected length ratio of 0.959 $\pm$ 0.037 on external test set (n=90). This indicates a high overlapped ratio between the AI's prediction and clinicians' annotation, and high capacities for extraction of primary airway branches. Although SABRE demonstrates superior performance in terms of DLR and DBR, it does not achieve the highest IoU. This is primarily due to some ground truth annotations missing the main bronchus. As a result, SABRE's predictions for some large bronchus, which are correct but not reflected in the ground truth, are regarded as false positives. The comparison results of SABRE and other models on different cohorts (COVID-19 and lung fibrosis) were shown in Table S2.

Additional results of SABRE's prognostic utility

The 1-year survival rate for the **STermAV** low-risk group was 30.8% (95% CI: 17.9% - 44.6%), compared with its medium-risk group's 15.6% (95% CI: 4.6 - 32.4%). The 1-year survival rate for the

SSmallAV low-risk group was 31.9% (95% CI: 18.7% – 46.0%), compared with its medium-risk group's 14.7% (95% CI: 4.1 – 31.6%). The 1-year survival rate for the **SMedAV** low-risk group was 28.6% (95% CI: 15.9% – 42.6%), compared with its medium-risk group's 17.7% (95% CI: 8.7 – 29.4%).

Algorithm 1. Pseudo-code of splitting airway segments

Input: Airway mask prediction $Y \in \mathbb{R}^{W \times H \times K}$

Output: Airway segments mask $Y' \in \mathbb{R}^{W \times H \times K}$

1. Localization of the main trachea T and remove it from Y (airway tree without trachea Y_{woT})

2. Localize the central point of the airway tree (the center of the last slice of trachea).

3. Split left and right airway trees and calculate their spatial center in the accumulated map along transverse plane and coronal plane.

4. Calculate the distance between the airway voxels and their corresponding tree centroids (for left and right airway trees separately).

5. Define the distance threshold to separate terminal, small, and medium trees. The thresholds for transverse plane are 25% and 55% distance percentiles.

6. Calculating the height of Y_{woT} and revise the segment label by its 3D spatial location.

The code is available at https://github.com/Nandayang/IPF-prognosis/tree/main

References

1. Pakzad A, Cheung WK, Van Moorsel CH, Quan K, Mogulkoc N, Bartholmai BJ, Van Es HW, Ezircan A, Van Beek F, Veltkamp M. Evaluation of automated airway morphological quantification for assessing fibrosing lung disease. *Computer Methods in Biomechanics and Biomedical Engineering: Imaging & Visualization* 2024: 12(1): 2325361.
2. Nan Y, Del Ser J, Tang Z, Tang P, Xing X, Fang Y, Herrera F, Pedrycz W, Walsh S, Yang GJIToNN, Systems L. Fuzzy attention neural network to tackle discontinuity in airway segmentation. 2023.
3. He K, Zhang X, Ren S, Sun J. Delving deep into rectifiers: Surpassing human-level performance on imagenet classification. In: Proceedings of the IEEE international conference on computer vision; 2015; 2015. p. 1026-1034.
